
JOURNAL INFORMATION
==============================
NLM Title Abbreviation: ScientificWorldJournal
Journal ID: ScientificWorldJournal
NLM Journal ID: 101131163
Journal ID: TSWJ

The Scientific World Journal

ISSN: 2356-6140
EISSN: 1537-744X
Publisher: Wiley

ARTICLE INFORMATION
==============================
PMCID: PMC6084197
DOI: 10.1100/tsw.2001.441
Article ID: 631241
Article version: 1
Subjects: Extended Abstract

Functional Characterisation of the CGRP Antagonist BIBN4096BS, in SK-N-MC Cells

Schindler Marcus
Doods Henri
Boehringer Ingelheim Pharma KG, Cardiovascular Research, 88397 Biberach, Germany
Publication date: 2001
Electronic publication date: 2001 May 1
Volume: 1
First page: 46
Last page: 46
Copyright: Copyright © 2001 Marcus Schindler and Henri Doods.
Copyright year: 2001
License: This is an open access article distributed under the Creative Commons Attribution License, which permits unrestricted use, distribution, and reproduction in any medium, provided the original work is properly cited.
License URL: https://creativecommons.org/licenses/by/3.0/

==============================
4th International Meeting on Calcitonin Gene-Related Peptide (CGRP)
*TheScientificWorld* (2001) 1(S1), 46
ISSN 1532-2246; DOI 10.1100/tsw.2001.441

### FUNCTIONAL CHARACTERISATION OF THE CGRP ANTAGONIST BIBN4096BS, IN SK-N-MC CELLS

Marcus Schindler and Henri Doods
Boehringer Ingelheim Pharma KG, Cardiovascular Research, 88397 Biberach, Germany

The 37-amino acid vasodilatory peptide calcitonin gene-related peptide (CGRP) has been implicated in the pathophysiology of migraine. We have recently developed a nonpeptide CGRP antagonist, BIBN4096BS, with subnanomolar affinity for primate CGRP-1 receptors. BIBN4096BS proved to be a valuable tool to dissect CGRP receptor pharmacology[1]. Interestingly, in functional assays in some tissue, the $pK_B$ values for BIBN4096BS differ depending on the agonist used, α- or β-CGRP[2]. We have therefore examined the potency of BIBN4096BS to block cyclic AMP accumulation caused by α- or β-CGRP, in SK-N-MC human neuroblastoma cells. We have also used a novel radioligand, $^{125}$I-Tyr$^0$-β-CGRP to determine the binding affinity of BIBN4096BS to probe whether a different β-CGRP receptor binding site exists.

$^{125}$I-Tyr$^0$-β-CGRP was custom synthesised by Biotrend. SK-N-MC were cultured as described[3]. Agonists ($10^{-4}$ to $10^{-11}$ M) were given simultaneously with BIBN4096 BS ($10^{-9}$ to $10^{-14}$ M), then incubated for 30 min at room temperature. Cyclic AMP was measured using Flashplates (NEN). Schild Plot analysis was carried out to determine $pK_B$ values (Graph Pad Prism). Radioligand binding on SK-N-MC cell membranes was carried out as described[3]. All experiments were carried out with $n \geq 3$, in triplicate.

BIBN4096BS blocked the cAMP stimulation evoked by α-CGRP with very high potency ($pK_B$ 13.4). BIBN4096BS was equally effective in blocking β-CGRP effects ($pK_B$ 13.7). The slopes for both Schild plots were not different from unity. In binding experiments, BIBN4096BS displaced $^{125}$I-Tyr$^0$-β-CGRP with an Ki value of 11.7, which is not significantly different to that previously determined using $^{125}$I-h -α-CGRP.

BIBN4096BS is a highly potent full CGRP antagonist on SK-N-MC cells. The equipotency of BIBN4096BS versus α-or β-CGRP suggest that there are no differential binding sites present in this cell line.

### REFERENCES

1. Wu, D. et al. (2000) Eur. J. Pharmacol. 400, 313–319.
2. Wu, D. et al. (2001) this meeting.
3. Doods, H. et al. (2000) Br. J. Pharmacol. 129, 420-423.
